# On the Safety of Implanted Breast Prostheses in Accidental Impacts

**DOI:** 10.3390/ma16134807

**Published:** 2023-07-04

**Authors:** Gerardus Janszen, Michela Arnoldi, Valeriano Vinci, Marco Klinger, Luca Di Landro

**Affiliations:** 1 Department of Aerospace Science and Technology, Politecnico di Milano, 20156 Milano, Italy; michela.arnoldi@polimi.it (M.A.); luca.dilandro@polimi.it (L.D.L.); 2 Department of Biomedical Sciences, Humanitas University, 20090 Milan, Italy; valeriano.vinci@hunimed.eu; 3 IRCCS Humanitas Research Hospital, 20089 Milan, Italy; marco.klinger@humanitas.it; 4 Department of Medical Biotechnology and Translational Medicine, Università degli Studi di Milano, 20126 Milano, Italy

**Keywords:** breast implants, silicone prosthesis aging, accidental impacts, impact/crash test, compression tests

## Abstract

The employment of breast silicone implants, both in aesthetic and reconstructive medicine, is widespread thanks to their recognized biocompatibility and durability. Some critical situations, for example, in the case of accidental impacts, may induce concerns by potential patients about their use. Dynamic tests reproducing frontal impacts at speeds up to 90 km/h, with anthropomorphic dummies carrying 330 cc prostheses and wearing safety belts, were conducted. Tests showed a significant probability of internal gel loss following implant damage at the highest speed. Moreover, considering that prostheses may remain implanted for many years, the effects of accelerated aging at 37 °C, 60 °C, 75 °C and 90 °C in physiological solution were also investigated. Tensile tests of the shell material and compressive tests of the full prosthesis showed evidence of variation in the prostheses’ mechanical characteristics after aging, which affects their stiffness, deformability and strength. These results stress the importance of medical investigations for possible damages of the implanted prostheses in the case of an accident.

## 1. Introduction

The use of silicone breast implants for aesthetic or reconstructive surgery dates back about 60 years. Since their invention by Cronin [1], it is estimated that far more than eleven million women have undergone breast implant insertion worldwide [2]. At present, although alternative materials have been proposed and used as breast prostheses, e.g., polyurethane foam-based implants, silicone prostheses remain the most employed solution in reconstructive interventions. As with any other surgery, breast prosthesis implantation may suffer post-operation complications; however, additional adverse reactions may even result over a long time, which may be caused by both patient conditions and/or the type of implant. A wealth of medical research has been dedicated to the reliability of different commercial prostheses so that the safe use of different types of implants, e.g., silicone gel filling vs. saline solution filling or textured vs. smooth shell surface, has been sometimes questioned over the years, with effects on the public perception about their safety and leading to oscillations in their market success [3,4]. Such controversial discussions have been mainly in relation to human-body compatibility and possible critical effects in medical terms. Breast Implant-Associated Anaplastic Large Cell Lymphoma (BIA-ALCL) or Squamous Cell Carcinoma (SCC) are relevant examples of such clinical effects, which have found great attention from medical researchers and institutions. BIA-ALCL, in particular, an uncommon T-cell non-Hodgkin lymphoma, has been the subject of extensive investigations over the last decade. It has been related to the surface texturing of implants, although the actual origin is still debated. It usually presents after years from implantation as swelling and/or pain in the breast area. When recognized, it often requires the removal of the implant; fatal consequences may result if not promptly diagnosed [2,3,4,5,6]. These findings led to the recall and retrieval from the market of some implant types and had significant effects on the public perception of their safety, inducing oscillations in their commercial success [3,4,7].

From a mechanical point of view, the danger of rupture of the prosthesis with inner filler leakage is one of the various possible complications related to the utilization of breast implants, which potentially interests the totality of implanted women, irrespective of their medical conditions. Thus, it has been the object of a number of investigations, mainly aimed at evidencing the possible clinical consequences and the most reliable methods to detect such damages. It is generally assumed that most traumas do not involve prosthesis rupture, except in the case of impacts with sharp objects. Among the different mechanisms proposed for implant rupture, the most common is the damage from surgical instruments (51–64% of all causes), followed by unidentified opening/rent without indication of cause (no evidence of sharp instrument damage, 35–37%) and fold flaw (8%). Shell swelling (reduction of the shell strength due to migration of the silicone fluid from the gel to the shell), delamination, manufacturing defect, surgical impact and trauma to the implant, such as external pressure to the chest or closed capsulotomy, represent the rest [8,9,10]. However, it should be considered that in the case of high-viscosity silicone gel filler, accidental ruptures may remain undetected for a long period of time with little or no sign for the involved person [11,12]. Ruptured implants may cause breast pain, shape changes, irritation or, in the worst cases, secondary effects such as cavitary lesion or pleural silicone collection [12]. Nonetheless, slow, silent perfusion of the gel in the surrounding body tissues may occur with adverse results not immediately evident, so that the actual ruptures may be possibly much more common than expected [13].

In this framework, the chance to investigate to what extent mechanical stresses due to fortuitous impacts occurring in human life, for instance, car collisions or accidental bad falls, may have critical results for implant integrity and can lead to probable important gel leakages of great relevance. Moreover, considering that the duration of implanted prostheses may exceed 30 years [14], it is important that, in addition to clinical compatibility, material aging effects over the mechanical failure probability are estimated. Previous studies have shown—in the restricted time/temperature range investigated—a significant, although limited, effect of aging over the viscoelastic behavior of general medical-grade silicone rubbers. On the other hand, few indications about the long-term effects of the ultimate mechanical properties were reported [15,16]. However, it should be underlined that most medical studies are dedicated to post-rupture effects, while engineering-based investigations about the conditions leading to possible implant failures are quite limited.

Previous indications—gathered from preliminary experimental tests with explanted prostheses—suggest that accidental impacts, such as those experienced in car crashes, can produce prosthesis rupture followed by relevant gel leakage. In addition, it was argued that shell mechanical response could be significantly affected by aging in the body condition. Whether gel swelling or silicone shell material aging should be considered the main cause of such changes is still under discussion [17,18,19]. Mechanical tests of shell material collected from implants removed after different time periods (up to 20 years) demonstrated the significant effect of aging on shell strength; the prosthesis type and implant history considered allowed the estimation of aging rate on a statistical basis [20].

In this paper, the results of crash tests with anthropomorphic dummies carrying prostheses and impacted at different velocities, representative of typical car accident situations, are presented and discussed. At the same time, a research program has been started to study the long-term, mechanical aging of initially new prostheses. At the global prosthesis level, squeezing load and failure pressure as a function of aging and body condition are being tested. At the shell material level, silicone shell strength, ultimate elongation and stiffness after accelerated aging at different temperatures and for different times are being characterized, with the goal of estimating the long-term variability of shell mechanical properties. A common approximation is based on the assumption that the rate of aging is increased by a factor of 2^0.1*ΔT^ for a ΔT increase in temperature, provided no additional chemical degradation reactions occur in the temperature range. With such an assumption, aging tests of one month at 90 °C, would correspond to about 40 months at 37 °C. More reliable models and predictions can be gathered if a set of results in a wide time–temperature range is available [15,16,21]. The results of aging tests up to 10 months in a wide temperature range are here reported and discussed; longer-term aging results will be later presented as soon as they become available.

## 2. Materials and Methods

New prostheses with a round shape and microtextured surface, produced by a primary manufacturer, were employed for static mechanical tests and aging tests. The prostheses consisted of a shell of medical-grade silicone rubber of about 0.5 mm thickness filled with a high-viscosity medical-grade silicone filling gel. For the shell material, a crosslink density of 1.3 × 10^−4^ mol/cm^3^ and a network chain mol weight of 8.6 × 10^3^ were indicated by the producer. Explanted prostheses not previously damaged were employed for car-crash tests. In this case, the aim was only to investigate the probability/possibility of having a rupture in the prostheses when subjected to the same loads that can derive from a car accident. No comparison between the new and explanted prostheses was investigated. These prostheses, produced by the same manufacturer, were also round, surface microtextured and explanted from a living patient for medical reasons not related to implant integrity. No evident damage was observed in these prostheses; the time lapse between insertion and explantation was between 12 and 18 months.

**Figure 1:**
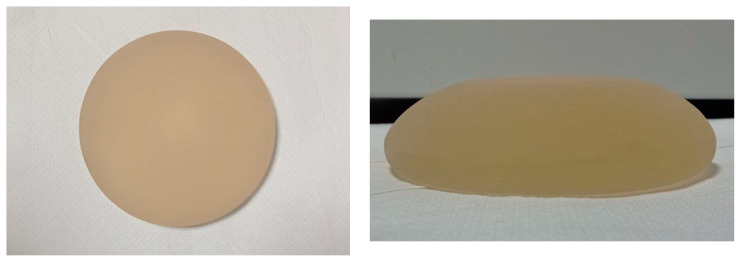
Compression-tested prostheses (volume: 275 cm^3^, diameter: 11.7 cm, projection: 3.7 cm).

Following a similar procedure previously set up with different used prostheses [19], squeezing tests were performed with new items. Two identical round prostheses of 275 cm^3^ volume (Figure 1) were compression tested between flat steel plates at a constant crosshead rate of 5 mm/min by an MTS858 Mini Bionix II (MTS Systems S.r.l., Torino, Italy) dynamometer. Compressive load, prostheses thickness and diameters were registered during the tests by a 15 kN load cell and video recording. The internal pressure was estimated as the ratio between load and contact area between prosthesis and compression plate. A first new, unaged item was pressed soon after the opening of the sterile, sealed packaging until rupture to estimate the rupture load and internal pressure. The second was pressed at 70% of the estimated failure load after aging at 37 °C in physiological saline solution (0.9%, NaCl). Results were determined for compression tests up to ten months of aging. Longer-term tests are underway.

**Figure 2:**
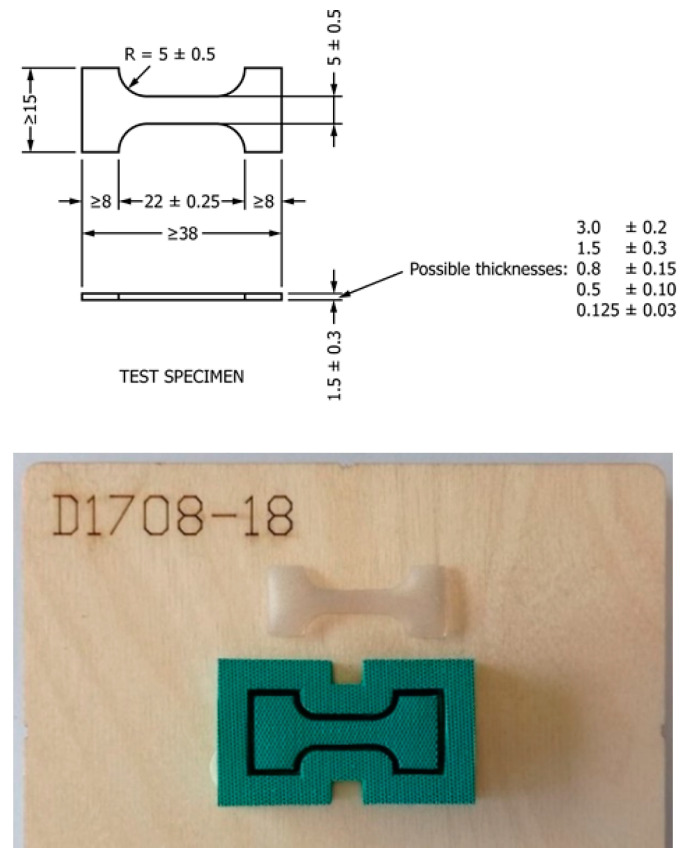
Microtensile specimen dimensions (in mm) and die-cutter for tensile tests.

To investigate the effects of time and temperature aging on the shell properties, three new prostheses from the same manufacturer and of the same type were opened with a blade and carefully cleansed with paper towels after complete removal of the internal gel. Microtensile specimens were cut from the upper part of the shell, with dimensions according to ASTM D1708 [22], by a die cutter (Figure 2). No specimens from the lower side of the prostheses were used since the thickness was consistently higher than the top side. Measurements were taken at different positions for each specimen with a micrometer gauge, indicating an average thickness of 0.521 mm ± 0.022 mm.

Materials implanted into the human body should maintain acceptable performance over their expected time of use. In vivo experiments may provide reliable results, but their time scales are often not sufficient for long-term predictions. Accelerated aging tests can be performed by conditioning the material at temperatures above the temperature of interest as a means of simulating the long-term aging process. On the basis of experimental data over a consistent time–temperature range, estimation models can be applied. Different empirical procedures and theoretical models, usually based on the use of the Arrhenius equation, have been proposed for medical-grade materials [15,16,22]. An accepted, simplified approach is that a temperature increase (DT) produces an increase in the aging rate by a factor f = 2^(DT/10)^ [23].

Different sets, consisting of 15 specimens each, were immersed in physiological solution maintained at 37 °C, 60 °C, 75 °C and 90 °C. Five specimens of each set were periodically extracted from the solution and tensile tested after aging (6 months, 1 year, 2 years) at each temperature level. According to the procedure proposed in ref [23], 2 years of aging at 75 °C would correspond to approximately 28 years at 37 °C. The complete set of results will be correlated and discussed with the aim of estimating a more reliable aging rate in body temperature conditions. In order to obtain a fast, preliminary indication of possible physical aging sensitivity, two subsets of five specimens, each from a different prosthesis of the same type, were maintained for one month and two months at 90 °C in saline solution. Unaged specimens and aged specimens were tensile tested at a 50 mm/min elongation rate up to failure with an INSTRON 4302 testing machine (1000 N load cell). Strength, elongation and stiffness were thus evaluated and compared. Analogous tests and evaluations were conducted for specimens maintained in saline solution at different temperatures that have reached the first period of aging (six months).

Crash tests to simulate car accidents were performed in a facility at the transport safety laboratory (LaST) of Politecnico di Milano. An anthropomorphic dummy (Hybrid II; H2-50) with round prosthesis applied over breast position by adhesive tape was placed on a seat and fixed by a four-point seat belt. The seat was mounted on the testing sled, which can be accelerated along an 80 m rail at the desired speed by a controlled pneumatic ram. Hybrid II dummy is regularly employed for the testing of aeronautic seats and vehicle safety belts. A decelerating/stopping system based on energy absorbers made of plastically deformable aluminum tubes simulates a crash; the selection of proper deformable absorber dimensions and thickness allows us to select a controlled deceleration program, which is recorded by accelerometers mounted on the sled. One test at 50 km/h and one test at 90 km/h impact speed were performed. The same absorbers, corresponding to a maximum deceleration of about 25 g, were selected for both impacts. The whole progress of acceleration and impact events were also recorded by a high-speed Phantom-V210 video camera. Visual inspection of prostheses after impact was carried out to evidence possible shell damage and/or relevant loss of internal gel.

## 3. Results

Following previous experiences [17,19], static compression tests were applied to record and characterize the response of breast prostheses up to silicone shell breaking. In particular, the tests allowed the estimation of the failure pressure of the new prostheses examined and the corresponding biaxial strength of the silicone rubber shell.

Static tests were carried out on two equal round-shaped breast implants with a volume of 275 cc, a diameter of 11.7 cm and a nominal side projection of 3.7 cm. One of the specimens was compressed till rupture, while the second was compressed after an aging period at 70% of the measured rupture load.

**Figure 3:**
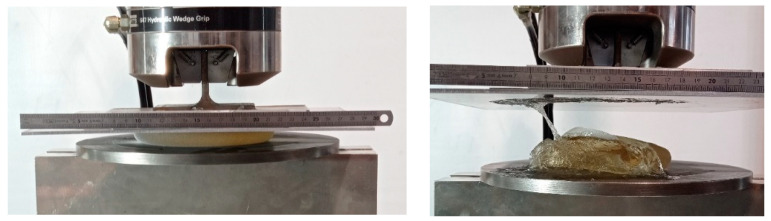
Compression test set-up.

**Figure 4:**
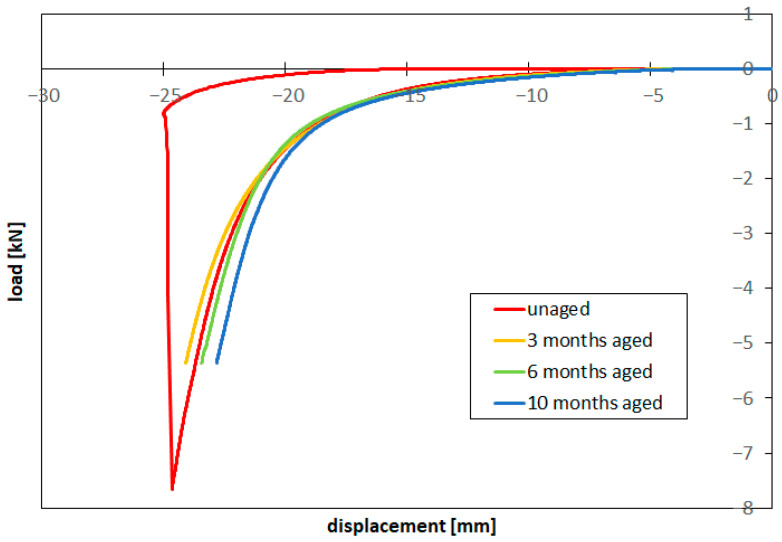
Uniaxial compression load–displacement curves for new (up to failure) and aged prostheses.

Figure 3 shows the compression set-up during the tests and at the end when the prosthesis shows clear rupture. Throughout the tests, load and plate position were recorded. The contact area between the deformed prosthesis and the plates at maximum load was also estimated by measuring the footprint area of the specimen on the plates to evaluate the internal pressure and stress at rupture. In Figure 4, the load–displacement curves are reported for the test on a new prosthesis and on a prosthesis aged in saline solution at 37 °C for three months, six months and ten months compressed up to 70% of the previously measured rupture load. After each test, the aged prosthesis was again placed in the heated bath to continue the aging process. The compression test was periodically repeated, with the same loading condition (up to 70% of rupture load) until failure, to verify the possible influence of aging on the overall behavior of the prostheses, in particular, over shell rupture strength and elongation.

The measured rupture load for the new prosthesis was 7650 N, reached at a final prosthesis thickness of 8.44 mm. The internal pressure at rupture was about 2.35 bar. It is also to be noted that for the new prostheses, rupture was reached at an internal pressure that was similar, but somewhat higher, to that (about 2 bars) previously measured with prostheses explanted from living patients [19]. Although, for the latter, the implantation time period was not known, it is likely that such difference is a consequence of aging.

Compression tests performed with new prostheses after short aging times (3, 6 and 10 months) at 37 °C in saline solution at 70% of the estimated rupture force did not show signs of failure. In these tests, the prosthesis was pressed at 5359 N, corresponding to a maximum estimated internal pressure of 1.76 bar, i.e., about 75% of the rupture pressure. The same prosthesis will be tested after longer time periods in the same aging conditions. Figure 4 shows the compression load–displacement curves recorded for new and three, six and ten-month-aged prostheses. A significant increasing trend of the load-to-deformation ratio with aging time can be observed as a consequence of an increase in shell stiffness and/or an increase in the elasticity of the filler gel.

**Figure 5:**
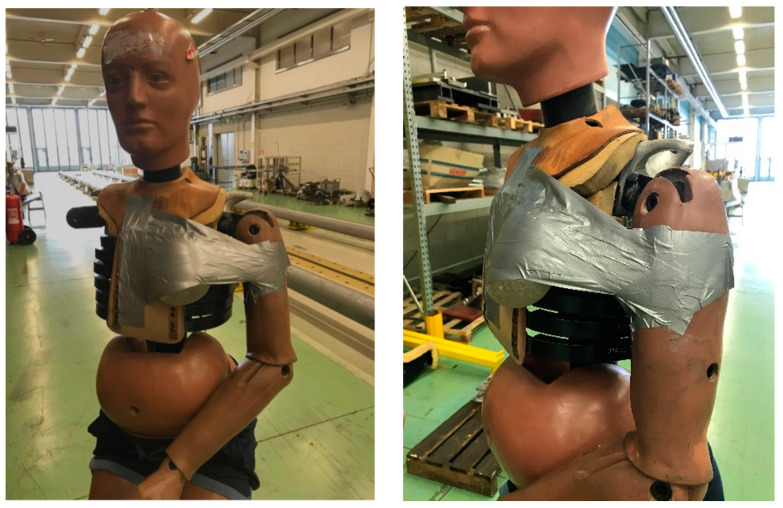
Assembly of prosthesis on Hybrid II dummy for crash tests.

Two dynamic tests were carried out on explanted round prostheses to reproduce car accident effects at different vehicle speeds. The prostheses with a volume of 330 cc were of the same producer and explanted from the same patient. The shell thickness, measured after the tests, corresponded (0.52 mm) to that of the new prostheses employed in static tests. The implants were fixed on an anthropomorphic dummy by adhesive tape in the exact location corresponding to actual implanted prostheses; of course, this arrangement is different but as similar as possible to that corresponding to a sub-glandular or sub-muscular insertion. A safety belt was positioned over the prosthesis. Figure 5 shows the prosthesis assembly on the dummy for the tests.

**Figure 6:**
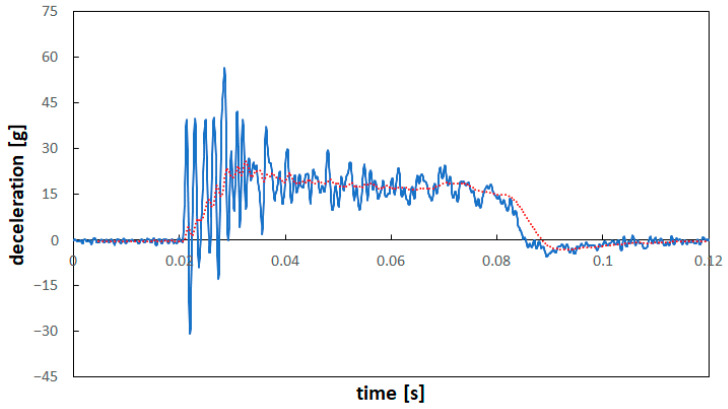
Deceleration vs. time of the crash event. Blue line: raw recording; red line: filtered results.

The recordings of the time history corresponding to the impact event for the two tests at 50 km/h and 90 km/h confirmed a maximum deceleration of about 25 g in both cases; this value is consistent with that experienced by a passenger in a vehicle during a frontal impact against a rigid barrier [24]. Figure 6 reports the deceleration vs. time results for the impact at 50 km/h; the blue line indicates the unfiltered signal from the accelerometer fixed on the sled, while the red line indicates the filtered result. It can be observed that after a peak corresponding to the onset of plastic deformation of the aluminum stopper, a fairly constant deceleration of about 20 g occurred during the whole pulse time. In case of impact at 90 km/h, as expected in consideration of the same stopper used, a quite similar deceleration peak was recorded, but with a longer pulse time.

**Figure 7:**
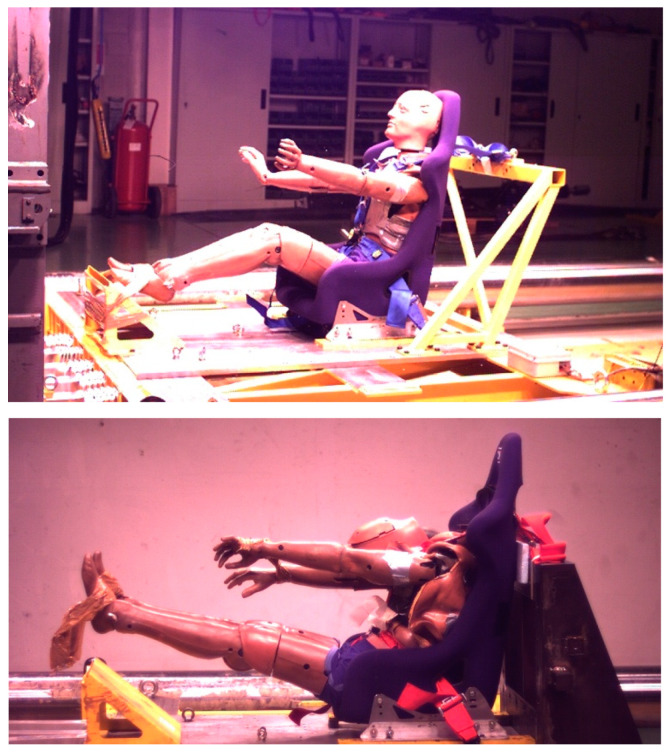
Instant frames of the sled/dummy during impact event.

**Figure 8:**
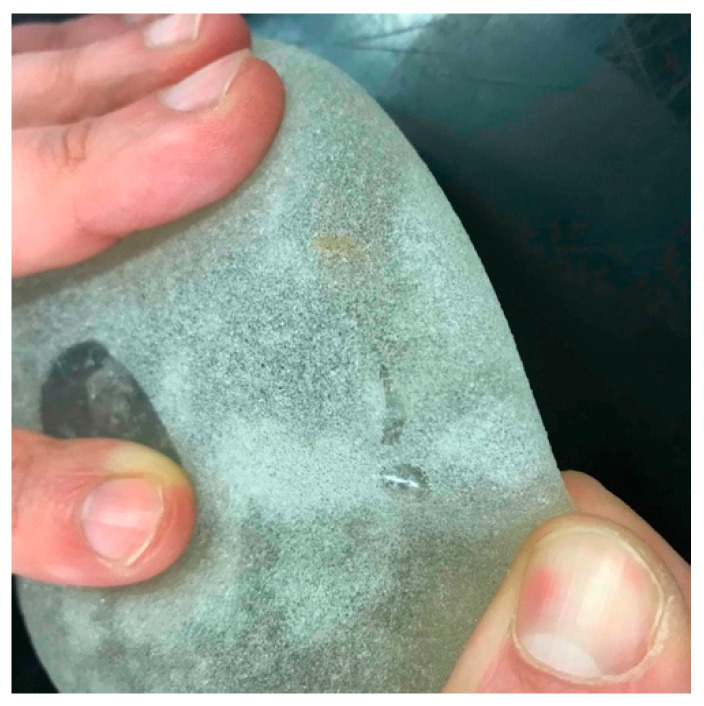
Rupture of the silicone shell and gel leakage after impact at 90 km/h.

Figure 7 shows two frames from the video recordings of the seat/dummy during the crash tests: a relevant compression of the prosthesis was evident. A visual inspection after the impacts showed that at the lower speed, no clear damage of the prosthesis was observed; on the other hand, after impact at 90 km/h, a rupture with some, although limited, internal gel leakage was clearly evidenced (Figure 8). The limited loss after impact, compared to the extensive loss observed in compression tests (Figure 3), is related to the very short time of the crash event.

## 4. Discussion

**Figure 9:**
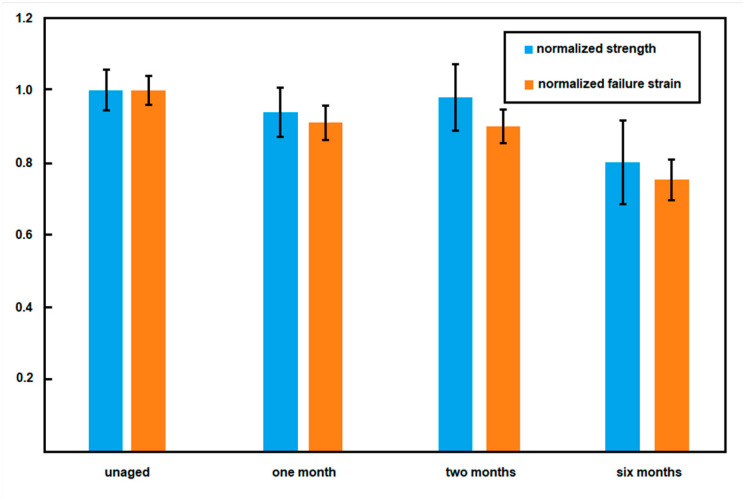
Decrease in strength and strain at failure during aging at 90 °C.

Preliminary tests of shell specimens after different aging conditions, i.e., new and after permanence at 90 °C in saline solution for one and two months, showed a significant reduction of failure elongation with aging and a corresponding variation of strength, notwithstanding the relatively limited exposure time. However, these results were later compared with those from specimens aged six months at 90 °C, which confirmed the decreasing trend of the material properties (Figure 9). Such variations suggest that high-temperature aging can produce physical changes in the polymeric elastomer, possibly with an increase in crosslinking density.

Table 1 reports the strength and strain at failure changes after different aging programs.

**Figure 10:**
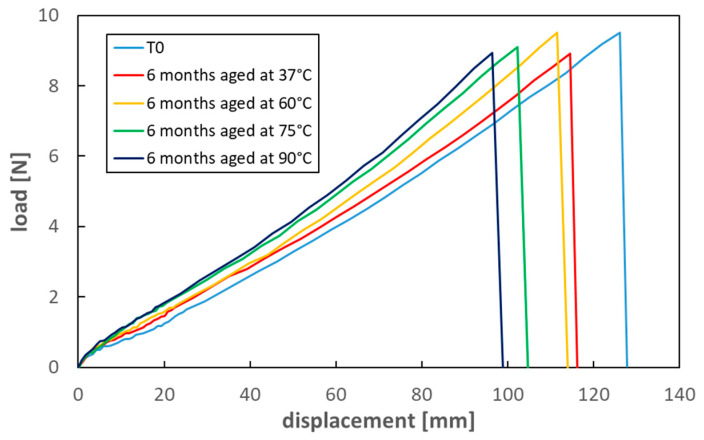
Tensile load–displacement curves for new and aged shell material.

A further confirmation of the mechanical response trend with time comes from the results of the six-month aging for the specimens maintained at four different temperatures: tensile tests showed an evident increase of silicone stiffness with aging time, which becomes more relevant the higher the temperature. Table 2 reports the strength and strain at failure values after six-month aging, while Figure 10 shows tensile load vs. displacement curves of aged silicone samples compared to unaged material.

Although the aging conditions here reported are only partially comparable with those relevant in real implants, these results again confirm that long-term physical and mechanical changes of shell response are to be expected. Since aging is still ongoing, such modifications will be better investigated and evaluated at longer times.

The recorded material changes, i.e., failure elongation reduction and stiffening, and their dependence on aging time and temperature, suggest that long-term aging can produce variations in the elastomer molecular structure, possibly consisting of an increase in crosslinking density.

The internal pressure estimated at rupture in uniaxial compression tests may be used as an estimate of all those cases where external pressure loads on the breasts are relevant, such as in high-depth scuba diving. In this situation, much depends on the location of the implants, which can be placed either behind the breast tissue, sub-glandular, or behind the pectoralis chest muscles, sub-muscular. Moreover, the maximum pressure load is significant with reference to potentially critical impacts with blunt objects, for example, in a car crash with the passenger wearing a safety belt over the breast or in the case of a person with implanted prosthesis falling flat on her chest. Such impacts may be significantly more severe than those prescribed by EN ISO 14607-2018 [25], which is the reference Standard for mammary implants, involving implant compression by a falling weight of limited energy.

Although the configuration adopted for the dynamic tests is typical for racing cars, breast injuries due to the pressure of seat belts are reported as not uncommon [26].

Of course, limited damage of the shell does not imply an immediate medical intervention, and the results here reported do not intend, by no means, to suggest avoiding the adoption of safety belts and airbags, which is certainly of paramount importance to prevent primary injuries to vehicle passengers in case of accidents [27]. On the other hand, secondary damages, which may produce long-term effects, cannot be excluded and should be considered in post-accident medical investigations by aware physicians. This recommendation becomes even more substantial with the aging of implanted prostheses, which can lead to significant changes in the mechanical response.

## 5. Conclusions

Breast implants for aesthetic or medical purposes have been widespread for many decades. As with other medical implants, their production and distribution have been regulated by specific requirements, mainly regarding their body compatibility and durability from a medical point of view. The chance of mechanical damages during their lifetime is, however, extensively reported and documented in the medical literature; moreover, indications exist, suggesting that actual silent damages are consistently more diffuse than expected [8,13]. In this scenario, situations can be assumed which suggest particular attention in relation to the safety of breast implants. Accidental events, such as car crashes or bad falls, are here confirmed as possible causes of significant damage to implanted prostheses. Crash tests showed that impacts at a vehicle speed of 90 km/h might be sufficient for damaging implanted prostheses. In addition, the possible long-term aging of the silicone shell, although of limited relevance in terms of body compatibility or reaction, can lead to some degradation of mechanical properties, rendering the outcome of accidental events even more worthy of attention. Results of accelerated tests at 90 °C suggest that aging can significantly reduce the overall extensibility of the silicone shell up to about 25% of the initial value, with an analogous yet lower effect over shell strength. An increase in stiffness is also evidenced. These results are consistent with an increase in the crosslink density of shell rubber due to aging. Further investigations are underway to better correlate the time vs. temperature aging effects; these will allow a quantitative estimate of the long-term aging at the body temperature. However, considering that prostheses may remain implanted for more than 30 years, similar modifications of mechanical response can be expected.

## 6. Limitations of the Present Study

In the literature, generic, unidentified damages consisting of opening/rent are reported as common without a more specific indication of a possible cause. In this paper, impact tests on two explanted prostheses confirmed the chance that a vehicle accident might lead to implant damage and loss of inner gel. A larger number of tests could certainly provide a statistical basis for further considerations in this regard. However, results obtained so far already suggested that a deeper investigation on the possible effects of aging of prostheses and the materials they are made of is of interest in view of their long-term life prediction.

Information about the effects of actual in-body aging over the response to mechanical impacts and on the overall properties of implanted prostheses can be gathered if a number of undamaged implants produced by the same manufacturer and of the same type/dimensions could be explanted after extended and exactly known periods of time. Such information is not available at the moment, but when collected, it could significantly improve the possible predictions about actual long-term expectations, integrating the results of the present study.

## Figures and Tables

**Table 1:** Mechanical degradation of unaged and aged shell material.

	Strength Decrease	Strain at Failure Decrease
Unaged	0%	0%
Aged—90 °C in saline solution (1 month)	6%	9%
Aged—90 °C in saline solution (2 months)	2%	10%
Aged—90 °C in saline solution (6 months)	20%	25%

**Table 2:** Mechanical properties of unaged and aged shell material.

	Strength (MPa)	Strain at Failure (%)
Unaged	4.88 ± 0.51	561 ± 31
Aged—37 °C in saline solution (6 month)	4.85 ± 0.64	545 ± 45
Aged—60 °C in saline solution (6 months)	4.71 ± 0.41	514 ± 26
Aged—75 °C in saline solution (6 months)	3.89 ± 0.29	443 ± 19
Aged—90 °C in saline solution (6 months)	3.93 ± 0.46	420 ± 24

## Data Availability

No data is available.
